# Genetic Engineering of Immune Evasive Stem Cell-Derived Islets

**DOI:** 10.3389/ti.2022.10817

**Published:** 2022-12-05

**Authors:** Sara D. Sackett, Samuel J. Kaplan, Samantha A. Mitchell, Matthew E. Brown, Adam L. Burrack, Shane Grey, Danwei Huangfu, Jon Odorico

**Affiliations:** ^1^ Division of Transplantation, Department of Surgery, UW Transplant Center, School of Medicine and Public Health, University of Wisconsin, Madison, WI, United States; ^2^ Developmental Biology Program, Memorial Sloan Kettering Cancer Center, New York, NY, United States; ^3^ Weill Cornell Graduate School of Medical Sciences, Weill Cornell Medicine, New York, NY, United States; ^4^ Department of Microbiology and Immunology, Medical School, University of Minnesota, Minneapolis, MN; ^5^ Center for Immunology, Medical School, University of Minnesota, Minneapolis, MN, United States; ^6^ Immunology Division, Garvan Institute of Medical Research, St Vincent’s Hospital, Sydney, NSW, Australia

**Keywords:** regenerative medicine, type I diabetes, allograft rejection, CRISPR, HLA allobarrier

## Abstract

Genome editing has the potential to revolutionize many investigative and therapeutic strategies in biology and medicine. In the field of regenerative medicine, one of the leading applications of genome engineering technology is the generation of immune evasive pluripotent stem cell-derived somatic cells for transplantation. In particular, as more functional and therapeutically relevant human pluripotent stem cell-derived islets (SCDI) are produced in many labs and studied in clinical trials, there is keen interest in studying the immunogenicity of these cells and modulating allogeneic and autoimmune immune responses for therapeutic benefit. Significant experimental work has already suggested that elimination of Human Leukocytes Antigen (HLA) expression and overexpression of immunomodulatory genes can impact survival of a variety of pluripotent stem cell-derived somatic cell types. Limited work published to date focuses on stem cell-derived islets and work in a number of labs is ongoing. Rapid progress is occurring in the genome editing of human pluripotent stem cells and their progeny focused on evading destruction by the immune system in transplantation models, and while much research is still needed, there is no doubt the combined technologies of genome editing and stem cell therapy will profoundly impact transplantation medicine in the future.

## Introduction

Diabetes mellitus is a complex metabolic disease which currently affects more than 30 million people in the United States and 463 million people worldwide with annual projections (1) indicated to continue to climb 2%–3% per year (2,3). Pancreatic islet endocrine cells are the major glucose and energy metabolism control mechanisms of the body. Despite continuing advances in insulin delivery technology and recombinant insulins, diabetes and its complications still claim the lives of millions of people as a result of ketoacidosis, hypoglycemic coma or chronic cardiovascular, eye, nerve and kidney damage (4). Existing beta cell replacement therapies, such as whole vascularized pancreas or islet transplantation, can achieve long-term normoglycemia and insulin independence in patients thereby forestalling end-organ complications. However, these therapies suffer from several key limitations. First, the shortage of organs make this option available to very few that fulfill the criterion, and second, the need for life-long immunosuppression to prevent allograft rejection. Severe complications related to immunosuppressive medication toxicities and chronic rejection continue to plague these approaches limiting their long-term success (5). An ideal β cell replacement therapy strives towards both generating an abundant supply of functional β cells *and* identifying a means to downregulate immune responses to suppress rejection and/or autoimmunity that is not associated with immunosuppression-related toxicities while prolonging graft function.

Human pluripotent stem cells have the potential to provide an unlimited supply of insulin-producing β cells for treating patients with diabetes (T1D, T2D, MODY, monogeneic diabetes). Human embryonic stem cell (hESC) lines, and human induced pluripotent stem cells (hiPSCs), which are generated by genetically reprogramming terminally differentiated somatic cells into a pluripotent state, have entered clinical trials to treat a multitude of disease from heart failure to macular degeneration, spinal cord trauma and diabetes, among others. Human iPSCs hold the additional potential for patient-specific therapies, thereby theoretically removing the necessity for immunosuppression. To date there have been advances in directing human pluripotent stem cells (hPSC) through stepwise differentiation protocols into functionally mature glucose-responsive and potentially therapeutic stem cell-derived islets (SCDIs). Progress from multiple groups and companies have contributed to the development and review of these protocols and advancements (6-20) and has led to the recent initiation of clinical trials (10-25) (ClinicalTrials.gov identifiers NCT02239354, NCT03163511, NCT02939118 and NCT04786262). Recent peer reviewed publications, as well as company reports, of first clinical experiences highlight proof of concept (10,23).

As hPSC-derived islets move into initial clinical trials, a number of factors could impact immediate and long-term success of this very young field, including off-target cells and the complex role of immunogenicity, among others. In this review, we will focus on immunogenicity-related issues of SCDI therapies. We will discuss mechanisms of islet destruction, and genome engineering strategies designed to impede alloimmune destruction. Additionally, we will discuss new advances in humanized animal models designed for studying the effects of these genomic perturbations on human immune responses to stem cell progeny. Lastly, we will discuss current approaches for developing genetic screens for identifying additional immune-protective genes.

## Mechanisms of Islet Destruction

Understanding the mechanisms by which the immune system reacts to and can destroy transplanted islets will inform efforts to subvert these pathways and prevent rejection of transplanted stem cell-derived islet organoids. Innate and adaptive immunity as well as autoimmune memory responses are all potential barriers in T1D recipients. While human islet and pancreas transplantation is successful with greater than 80% of patients achieving short-term insulin independence, long-term success requires powerful, continuous immunosuppressive medications. Underscoring the clinical challenge, Human Leukocyte Antigen (HLA)-identical transplants may succumb to recurrent autoimmune destruction (26,27). Knowledge of the mechanisms of islet transplant rejection and autoimmunity largely derive from rodent studies; several excellent recent reviews update our knowledge in this area (28-30). Briefly, as autoimmunity in rodent models of type 1 diabetes requires both CD4 and CD8 T cells (31), autoantigen expression is required for graft infiltration by autoreactive CD8 T cells following syngeneic islet transplantation (32) and rejection of vascularized organs appears CD4 T cell-dependent (33) it is probable that both T cell subsets contribute to the combination of autoimmunity and alloimmunity that would occur following implantation of genetically-disparate or genetically engineered insulin-producing cells into an autoimmune recipient.

A potential opportunity for novel intervention relates to the innate immune instant blood mediated inflammatory reaction (IBMIR). IBMIR represents a key factor in the immediate loss of Islets transplanted into the liver and is currently managed with anti-coagulant and anti-inflammatory medications. A key molecular step in IBMIR is islet expression of tissue factor (TF) (34,35). TF expression is regulated by the pro-inflammatory transcription factor NF-kB following exposure to cytokines as well as by the activated inflammasome (36). Thus, engineering stem cells to be non-responsive to inflammasome activation, to be less sensitive to NF-κB activation, or to lack TF itself could be beneficial. However, to what extent SCDIs elicit IBMIR, express TF and/or are protected by cytokine inhibitors has not been studied despite ongoing clinical trials studying stem cell-derived islet transplantation into the liver. In addition, due to expression of ABO antigens on tissue cells, solid organ transplants and islet transplants must obey ABO compatibility, but whether this is true for SCDIs is unknown. Also, it is unknown whether SCDIs which generally contain immature and mature cell types, express high levels of ABO glycoprotein antigens.

Innate immune mechanisms include recruitment and activation of natural killer (NK) cells which offer further opportunities for enhancing islet resistance to immune attack. As NK cells are activated by ligands expressed on ischemically and mechanically damaged isolated islets, these ligands would represent promising candidates for gene editing SCDIs, but to what extent NK cells would be activated by SCDI grafts remains to be determined. Furthermore, editing NK ligands may promote NK cell-mediated attack as NK cells execute the “missing self” response, i.e., rejection of Major Histocompatibility Complex (MHC) Class I deficient or non-self MHC Class I expressing allografts. To overcome this problem some groups are engineering expression of non-classical HLA-E, HLA-G or CD47 into stem cells or rodent islets(37-39).

The cellular adaptive immune response is primarily mediated through alloreactive host T cells. Host T cells can be activated *via* multiple mechanisms including by 1) interaction of their T cell receptor with intact allogeneic MHC on donor cells (direct pathway), 2) donor peptides presented by self-MHC on recipient antigen presenting cells (APCs) (indirect pathway), or 3) through recognition of allogeneic MHC displayed on recipient APCs after their transfer *via* cell-cell contact or through extracellular vesicles (semi-direct pathway, MHC cross-dressing) (40-43). Each of these T cell activation pathways requires specific steps that provide unique opportunities to engineer resistance into stem cells. Common steps in T cell activation include a requirement for co-stimulation and other reinforcing positive signals, as well as an absence of inhibitory signals, from antigen presenting cells. Studies in mice and humans show that co-stimulatory blockade with CTLA4Ig (abatacept), or analogs such as Belatacept, effectively inhibit cytotoxic T cell responses and prolong islet allograft survival but requires adjunctive immunotherapy (reviewed in (44)). Forced expression of cytotoxic T lymphocyte antigen 4 (CTLA4), or the programmed cell death (PD) molecules PD-1/PD-L1 in SCDIs could short circuit T cell activation and facilitate immunosuppression-free survival. However, there are many aspects to these processes that are not fully understood with regards to SCDIs. Whereas cadaver islets contain dendritic cells (DCs) acting as professional APCs potentially seeding direct alloresponses, SCDIs may not contain this population, or endothelial cells which can also express MHC following inflammatory signals, and therefore indirect or semi-direct responses may predominate influencing the choice of inhibitory molecules to be targeted in SCDIs.

New discoveries in mechanisms of immune homeostasis also provide new avenues for SCDI engineering. The ubiquitin editing enzyme A20, encoded by *TNFAIP3*, and FasL have been shown to play a dominant role in protecting islet allografts (45,46) together with a short course of rapamycin. A20 overexpression inhibits the expression of inflammatory mediators and raises inflammatory signaling thresholds which promotes the development of antigen specific Tregs supporting immune tolerance and islet survival (45). Another approach, FasL coating of islets or embedded in microgel with islets in conjunction with subtherapeutic rapamycin also promoted long-term allograft acceptance in rodents and non-human primates related to Treg induction (47,48). Stromal cell-derived factor 1-alpha, aka CXCL12, was also shown to promote islet allo and xenograft survival through multiple postulated immune regulatory mechanisms (49,50). Thus, these molecules could be tested in overexpression models of genome editing of SCDIs.

Though the major pathways of islet rejection are not fully understood, and may differ substantially between rodents and humans, the information we do have provides a rich source of opportunities for experimental interrogation of protecting SCDIs from cellular mechanisms of innate, adaptive and autoimmune mediated destruction.

## Immunogenicity of Stem Cell-Derived Pancreatic Lineage Cells

While undifferentiated stem cells maintain some level of immune privilege (51-53), they become recognized or visible to the immune system once differentiated. Therefore, development of strategies to avoid recognition of cells by the immune system and ultimate destruction will be critical to therapeutic effectiveness.

In mammalian systems every nucleated cell is adorned with cell surface antigens (54). In humans the genes responsible for these marker molecules are encoded by HLA genes. HLA genes are grouped into class I (HLA-A, -B and-C and less polymorphic -E, -F and -G), class II (HLA-DR, -DP, -DQ, -DM, -DN and -DO) and III (the complement cascade); HLA-A, -B, -DR, -DP, and –DQ are the most studied and important contributors to allorejection.

Studies have begun to interrogate the immunogenicity of SCDIs. While undifferentiated hPSC have low levels of MHC expression, leading to their evasion, as these cells differentiate the MHC signature is upregulated thereby increasing their vulnerability and exposure to the immune system (52,53). Similar to native human β cells, SCDIs express HLA Class I antigens which can be upregulated by cytokine exposure (55,56). However, while normal human β cells upregulate all MHC isotypes, gene expression profiling on the SCDIs revealed HLA-C to be predominantly expressed, a finding that may be due to the immaturity of the SCDIs (56). Interestingly, both stem cell-derived pancreatic progenitors and endocrine cells express complement inhibitory receptors, CD46, CD55 and CD59 (55). Additionally, it has been shown that human β cells upregulate PD-L1 when exposed to proinflammatory cytokines (57,58). Castro-Gutierrez et al. went on to show that while human primary β cells respond to inflammation by upregulating PD-L1, they found that their SCDIs did not (56); which is different from what Yoshihara et al. demonstrated (59).

Like human islets, SCDIs are vulnerable to alloreactive cytotoxic T lymphocyte (CTL) killing *in vitro* (55). In addition, preproinsulin (PPI)-specific CTLs recognize and kill SCDIs in the context of PPI peptides (55,56), similar to normal human β cells (60). SCDIs are similarly vulnerable to antibody dependent cellular cytotoxicity, but may be resistant to complement mediated cytotoxicity *in vitro* (55). Through genetic modification to introduce inducible PD-L1 expression, Castro-Gutierrez et al. showed that PD-L1 overexpression and HLA Class I knockout abrogated diabetogenic CD8 T cell activation (56). Collectively, these studies begin to define the immunogenicity of SCDIs.

## Methods of Genetic Engineering

Precise and efficient genetic engineering leverages targeted DNA double strand breaks (DSB) to potentiate desired editing. CRISPR-Cas9 tools have shown wide utility and complement editing systems like ZFNs and TALENs to enable knockout (KO) and knock-in (KI) of transgene cassettes, tags, and patient risk variants (61). For example, gene editing has been used in hPSCs to show that a noncoding variant downstream of *GATA6* affects *GATA6* expression and pancreatic differentiation, suggesting that this minor allele variant acts as a genetic modifier of the neonatal diabetes phenotype in patients with *GATA6* heterozygous mutations (62). Similarly, we have applied CRISPR-Cas9-mediated gene editing to recreate patient-specific missense mutations in *GATA6* and *NGN3* or *NEUROG3* for investigation of neonatal diabetes and pancreatic differentiation phenotypes (63,64).

KO and KI are keystone capabilities for engineering an immune privileged and safe beta cell therapy, but off-target editing effects and the proximity requirement to the DSB site for precise editing have limited the utility of early CRISPR-Cas9 systems. Many methods are being developed to overcome these limitations such as optimized Cas9 enzyme designs and new fusion constructs like those used in base editing (65). For instance, Cas9 nickases, variants of the Cas9 enzyme, were designed to cleave only one strand of the DNA to minimize off-target DSBs and subsequent undesired editing. Cleaving both DNA strands using a Cas9 nickase and two proximal gRNAs shows low off-target effects and allows efficient and complex editing in human iPSCs (66). An exciting recent expansion of this technology is prime editing, where, a Cas9 nickase is fused to a reverse transcriptase, and combined with a clever prime editing guide RNA design, allows precise nucleotide alterations that can be over 30 bp from the PAM site mitigating the proximity requirement (67,68).

The flexibility of CRISPR-Cas9 and new Cas9 variant-based editing tools can change the stem cell derived beta cell therapy landscape by supporting simple and robust manufacturing pipelines. Recent therapeutic efforts have largely taken allogeneic approaches that require only a single edited stem cell line to be produced and validated (e.g., ClinicalTrials.gov NCT05210530). Increased versatility and efficiency of genome editing technologies may enable allogeneic therapies with complex engineering that improves immunogenicity profiles through gene editing. In addition, a proof-of-principle study involving correction of an inherited mutation in the insulin locus also suggests feasibility of autologous therapies with personalized gene correction (69).

## Genetic Modifications Leading to Reduced Alloimmune Destruction and Increased Survival of Stem Cell-Differentiated Cells and Their Derivatives

**FIGURE 1:**
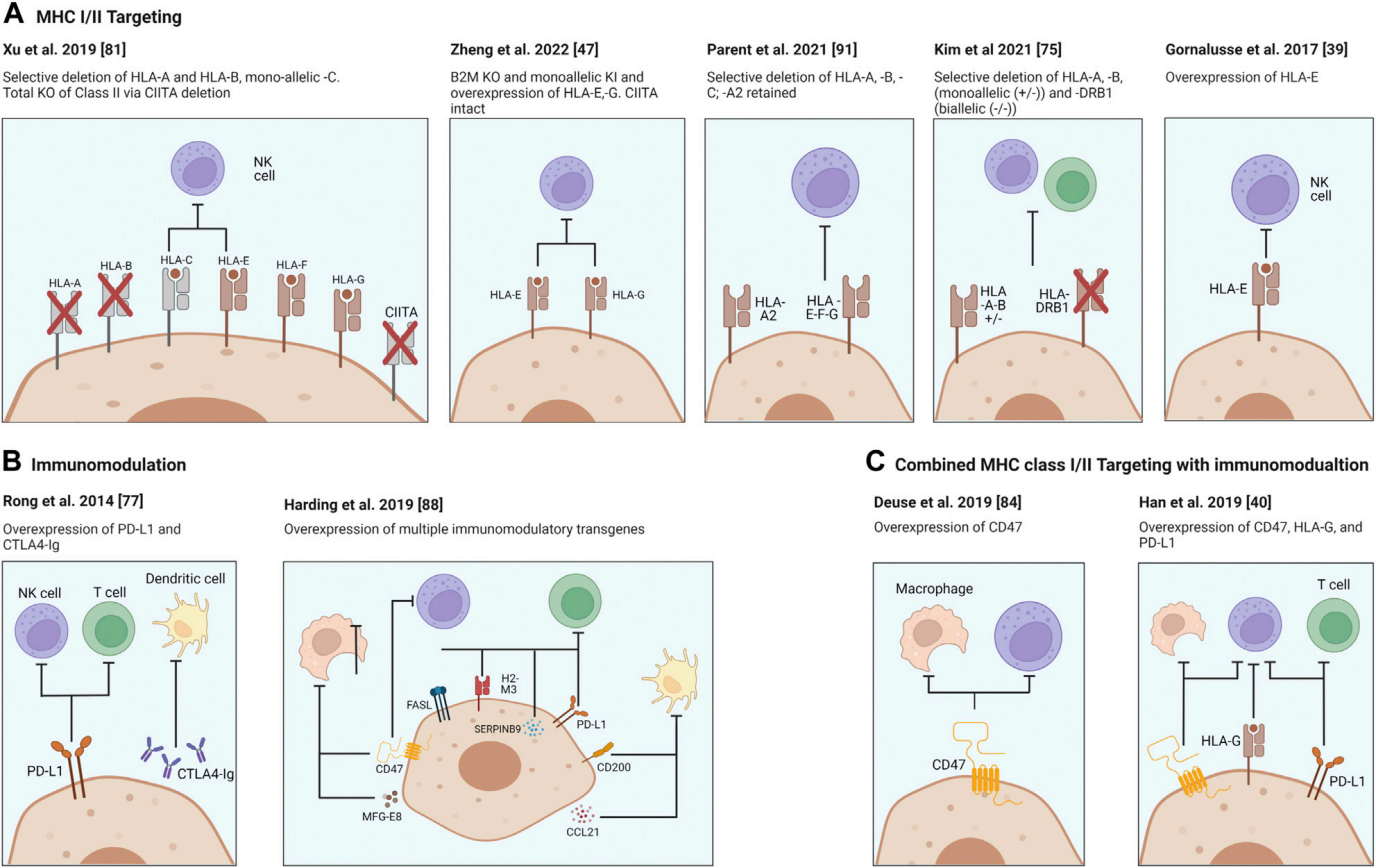
Strategies for providing immune protection of SCDI. Genome engineering of immune check point molecules and/or *via* HLA Class I and II surface molecules. Targeting B2M in HLA-I causes the disruption of expression of all class I genes, major A-C and minor E-G. Surface presentation of HLA Class II molecules is disrupted *via* knocking out the transcription factor CIITA. Figure was produced using Biorender (biorender.com).

For broad clinical use of stem cell-differentiated cells, it is imperative to reduce the alloimmune destruction after transplantation, if universal stem cell lines are to be more effectively utilized. Thus, a major active goal in the field is the development of compatible hypoimmunogenic cells which evade the immune system and reduce or eliminate the requirement for life-long immunosuppressive regimes while restoring tissue/cellular function. These issues have been addressed in several recent reviews and several approaches depicted in Figure 1 (70-74).

In order to utilize the power of stem cells the host immune response needs to be addressed. Disruption of β-2 microglobulin (B2M) interrupts surface presentation of MHC class I molecules encoded by HLA-A, -B, -C, -E, -F, and -G and prevents activation of CD8^+^ cytotoxic T cells. On the other hand, disruption of Class II transactivator (CIITA), a master regulator responsible for expression of HLA Class II genes, reduces antigen presentation to host CD4^+^ T cells. Each or both may be inactivated, and HLA class I and/or II knockouts may be coupled with the overexpression of immunomodulating transgenes. Two well characterized molecules, PD-​L1 and CTLA4-Ig are two immune checkpoint proteins being employed and ectopically expressed to protect the cells from the host’s T cells (75). Rong et al. have shown that these modifications prevented allogeneic rejection of teratomas in a humanized mouse model through disruption of T cell co-stimulatory and enhancing inhibitory pathways, both of which were necessary for allowing teratoma formation in their model (76). While promising this approach did not interrupt the MHC expression thereby potentially leaving engrafted cells vulnerable to allorecognition by the adaptive immune system.

However, complete removal of MHC Class I expression does not protect cells from NK cell attack and lysis but rather may activate them due to the “missing-self” response (77,78) and additionally may leave cells vulnerable to bacterial and viral infection due to inability to present these antigens to the host immune system. Addressing this concern, it has been demonstrated that it is possible to achieve alloimmune graft acceptance through genetic modifications, such as “knockins” and constitutive expression of immunomodulatory factors (Figure 1). To this end, Gornalusse et al. developed a B2M-HLA-E (a minimally polymorphic) fusion protein after complete B2M deletion in hPSCs (38) while Shi et al. similarly expressed a B2M-HLA-G fusion construct to stabilize the MHC and allow cell surface expression in B2M KO hPSC cells and showed hypoimmunogenicity and reduced NK-cell activation (38,79). This modification has the benefits of protecting stem cell derivatives from CD8^+^ T cell targeting and from NK-mediated cell lysis. Importantly however, not all NK populations may be affected due to differences in membrane receptor presentation, such as NKG2A, KIR2DL4 and ILT2. Another example is that of Xu et al. who derived iPSCs with disruptions in HLA-A/B but retained HLA-C expression and could demonstrate CD8^+^ T cell and NK cell evasion, although HLA-C presence may still allow presentation of bacterial and viral antigens (80). Other groups have also observed reduced NK cell activation upon non-classical MHC expression such as HLA-E and HLA-G. Zheng et al. found lentivirus overexpression of HLA-E and HLA-G in mesenchymal stem cells could prevent activation of the three major subtypes of NK cells (46). Lentiviral overexpression of a single-chain HLA-E was also used by Hoerster et al. to reduce allogeneic T cell proliferative and activation responses to *B2M* KO NK cells in co-culture assays (81). Taken together these approaches demonstrate methods to overcome NK cell “missing self” induced fratricide of KO somatic cell transplants.

Taking advantage of our knowledge regarding cancer cell survival pathways (82), another study looked to reduce NK cell activity through the overexpression of the transgene CD47, which is a ubiquitously expressed immunomodulatory suppressive gene (83,84). Deuse et al. demonstrated that CD47 was very effective at inhibiting NK cells and macrophages from killing MHC-deficient iPSCs in immunocompetent mice and report that these inhibitory signals are accomplished *via* an essential interaction with the signal-regulatory protein alpha (SIRPα). They further showed that blockade of the CD47 receptor renders the cells susceptible to NK cell killing.

Additionally in 2019 Han et al. sought to develop a strategy which addresses both adaptive and innate immune responses through genetic modifications to knockout the MHC class I and II expression followed by knock-ins (KI) to express the immunomodulatory factors PD-L1, CD47 and HLA-G (39). Of note, HLA-G is expressed during pregnancy at the maternal-fetal interface and is an NK cell inhibitory ligand (85,86). This study demonstrated that these modifications led to significant reduction in immune responses with respect to T cell, NK cell and macrophage-mediated killing *in vitro* assays.

While most studies focus on deletion of HLA-encoded MHC surface molecules, a study from Andras Nagy’s group targeted the upregulation or over-expression of additional immunomodulatory factors, CCL21, PD-L1, FASL, Serpinb9, H2-M3, CD47, CD200 and MFGE8 in mouse embryonic stem cells (87). These factors individually target specific cell subsets of the immune system or act on different mechanisms, and therefore could act synergistically. For example, CCL21 encodes for a cytokine that recruits activated dendritic cells. PD-L1, FASL, Serpinb9, H2-M3 target T-cells and NK cells. CD47 and CD200 prevent phagocytosis and MFGE8 can push macrophages towards an anti-inflammatory state. Multiple clones were generated exhibiting different degrees of over expression of each protein and two optimal expressing closes were tested for survival after transplantation as undifferentiated cells in a variety of immunocompetent mouse strains. It was shown that the expression of these factors allowed transplanted cells to survive and form teratomas, without any intentional modifications of the MHC locus. While the aforementioned studies focused on achieving reduced alloimmune responses to non-islet hPSC-derived cell types, such as undifferentiated cells, cardiomyocytes, endothelial cells, hematopoietic cells and retinal pigment epithelial cells, in different studies, it remains to be confirmed whether such approaches will be as effective for SCDIs.

## Genetic Modifications Leading to Reduced Alloimmune Destruction and Increased Survival of Stem Cell-Differentiated Islet Cells

Paving the way for the future possibility of allogeneic SCDI transplantation without immunosuppression, there has been significant progress towards improving immune evasion through genetic modifications (88). *B2M* knock-out aims to reduce T cell activation by preventing stable MHC class I formation on the SCDI cell membrane. The role of MHC class I in the SCDI—T cell interaction was explored through a set of *in vitro* orthogonal approaches: a trans-well assay, antibody blocking of MHC class I, as well as genetic KO of *B2M* which resulted in decreased CD25 and CD69 expression in the responding CD8^+^ T cell population (89). An alternative approach to improve immunocompatibility is PD-L1 overexpression, which was shown to dramatically improve SCDI functionality in a PBMC-SGM3 humanized mouse model, suggesting a measure of protection from alloimmune recognition (59). Notably, induction of endogenous PD-L1 through IFNγ pre-treatment of SCDIs also conferred protection upon transplantation to immune-competent mice implying a measure of protection against xenorejection. Although the transplanted cells were shown to regulate blood glucose out to 50 days post-transplantation, long-term time points were not included and could be of interest to characterize (59). In a separate study, PD-L1 overexpression in SCDI, achieved through an integrated inducible cassette, decreased IL2 secretion by diabetogenic TCR-expressing T cells (56). When further combined with a frameshift mutation in *B2M*, T Cell IL2 secretion was nearly abrogated, demonstrating the promise of multiplex editing involving MHC class I interference and PD-L1 overexpression (56).

MHC class I disruption is a major contributor to preventing T cell activation, but as mentioned in Section E, fully disrupting MHC class I surface expression may be associated with somatic cell graft lysis by NK cells (77,78). To address this concern, CRISPR-Cas9 was used in hPSCs to KO the polymorphic *HLA-A, HLA-B*, and *HLA-C* class I genes as well as MHC class II transactivator *CIITA* but retain the highly prevalent allelic variant *HLA-A2* and the other non-classical, less polymorphic *HLA-E, HLA-F,* and *HLA-G* genes that may protect cells from NK cell-mediated lysis (90). Co-culture of edited SCDIs with peripheral blood mononuclear cells (PBMCs) reduced CD107a (LAMP1) activated subset of NK cells and significantly improved survival following transplantation into immunodeficient mice which had been reconstituted with PBMCs from an HLA-A2^+^ donor. The retained HLA-A2 is proposed as the factor that enables HLA-E expression upon IFNγ stimulation, as a failure of HLA-E expression in HLA-ABC^null^ cells was restored upon introduction of HLA-A2-derived signal peptide. A complementary approach to combinatorial KI has been to discover and functionally characterize SCDI ligands that activate NK cells (91). Expression data suggested CD226 ligand PVR (CD155) and a co-stimulatory molecule of CD337 ligand B7-H6, B7-H3, to be promising NK activation candidates. While co-culture of *B2M* KO human SCDIs with human CD16_dim_ NK cells caused ∼80% of SCDI cells to become necrotic, co-culture of *B2M*, *CD155*, and *B7H3* triple KO SCDIs resulted in ∼20% necrotic cells, indicating a measure of protection from NK lysis. Triple KO pancreatic progenitors were then subcutaneously transplanted to NSG mice. Within 72 h of human NK cell injection, luciferase signal from *B2M* KO cells was markedly reduced, but triple KO cells showed similar survival to WT and to *β2M* KO HLA-E overexpression pancreatic progenitors. Collectively, these studies highlight the value of investigating how immune cells subsets interact with transplanted cells and chart a path towards generating hypoimmunogenic and universal cell lines for allogeneic stem cell therapies.

Genetic engineering is a promising avenue for overcoming survival challenges post-transplantation, and looking forward, multiplex editing may advance SCDI therapies that do not require immunosuppression.

## Modeling the *In Vivo* Immune Response to PSC Therapies

There is a critical need for assessing the *in vivo* immune response to PSC-based therapies prior to clinical trials. Human immune system (HIS) humanized mice offer a tractable pre-clinical *in vivo* model of the human immune response and have been used for a variety of transplantation immunology studies (92-95). There are a variety of HIS models available (96), but most useful for PSC transplant immunology studies are those models which incorporate both the infusion of human hematopoietic stem/progenitor cells (HSPCs) as well as thymic fragments into immune-deficient mouse strains to provide T cell developmental cues in the animals. The bone-marrow-liver-thymus (BLT) model (97) and NeoThy model (98) are two leading HIS iterations. Both harbor *de novo* generated human MHC-restricted T cells, and a complement of other adaptive and innate immune cell types useful for assessment of transplantation tolerance and rejection.

The BLT model has garnered concern over the immature nature of the fetal immune systems in the animals, in particular the naïve (99) and regulatory T cell subsets (100), spurring a search for higher-fidelity modeling of adult human immunity. We developed the NeoThy model using neonatal, instead of fetal, HSPCs and thymus in order to evaluate the impact of more developmentally mature tissue on the resultant immune cell repertoire function. Importantly in HIS models, not only does the humanizing tissue directly impact T cell development and function, but also the choice of immune-deficient mouse strain will impact the character and phenotype of accessory lymphoid and myeloid cells that develop, as will the method of myeloablation used for human HSPC engraftment (101).

Recently, immune-deficient mouse strains such as the NSG or NOG have been modified to improve human cell engraftment (102). Various groups have introduced mutations to these strains that obviate the need for irradiation-based myeloablation (103), for example, as well as adding human transgenes such as GM-CSF and IL3 that support a more-robust myeloid immune compartment (104), and therefore presentation of alloantigens.

Assessment of transplant rejection in HIS humanized mice can be determined by examining immune infiltration, activation and/or cytokine release post-transplant. To date, the humoral immune responses in these mice has been suboptimal, notably, with a lack of antibody class switching and T cell-dependent antigen responses to vaccination(105). Therefore, cellular immune responses are the primary focus until improved iterations of HIS mice can be developed.

Non-human primates (NHPs), such as rhesus macaques, currently play an important role in pre-clinical PSC studies (106,107). NHPs are useful for evaluation of human PSC-based therapies and associated immunosuppression requirements (108), as well as useful for gene editing studies (109). We recently developed a BLT-type “primatized” mouse model (110) for evaluation of the NHP immune response prior to conducting full-scale large animal studies. Experiments are ongoing to evaluate PSC cellular therapies in these primatized mice as a method to screen potential therapeutic and genetic modifications. Ethical considerations may prevent use of NHP primatized mice, as well as conventional HIS BLT mice using human fetal tissues.

A key consideration for the choice of *in vivo* model is the genetic composition of the immune system and humanized mice offer a unique opportunity to select humanization donors of particular genetic backgrounds. There are conflicting reports regarding the concept of autologous self-tolerance to iPSCs and/or their differentiated products (111,112,113) and it is possible to reconstitute a humanized (114) or primatized mouse model with an autologous immune system to test the hypothesis that an autologous graft will be tolerated as self. Importantly, the pathological target of a PSC therapy, will require careful consideration of the humanizing tissue source, especially in cases of autoimmunity e.g., T1D.

## Future Prospects: Genetic Screens

Genetic engineering tools also impact discovery efforts for stem cell derived β cell replacement. The progress of utilizing genome editing in hPSCs to create SCDI for transplantation without immunosuppression also points to the need to discover additional targets for gene editing to further improve engraftment and delay (or prevent) immune rejection. Genome-scale CRISPR screens have emerged as a powerful tool to address this need. In addition to CRISPR-Cas9 screens that we and others have performed to identify genes involved in the step-wise differentiation from hPSCs to insulin-secreting β cells)(115,116,117,118) recent studies have leveraged CRISPR screens to directly uncover immunomodulatory factors that mediate SCDI survival post-transplantation. For these experiments, a pool of cells is created where each cell has a different gene knocked out. Following an assay (ex. transplantation), the impact of knocking out every gene on a readout (ex. survival) is revealed. The first such screens were conducted in the mouse NIT-1 β cell line to uncover genes, which when mutated, would confer a survival advantage upon transplantation into a T1D mouse model(119). While most cells were destroyed upon transplantation, the authors collected the surviving cells and found that knockout of *RNLS*, a gene previously associated with autoimmune diabetes, protected cells from destruction through reduced stimulation of autoreactive CD8^+^ T cells and increased resistance to ER stress. Furthermore, *RNLS* KO β cells differentiated from hPSCs had increased protection from ER stress, reproducing an important finding from the mutant mouse β cells. A limitation of conducting screens using the mouse system is that there are known differences between mouse and human β cells and immunological contexts(120). Addressing this limitation, a human SCDIs transplantation survival screen has also been conducted(121). Human SCDIs were transplanted into Hu-PBL-NSG-MHC^null^ mice that also received human PBMCs. SCDIs were harvested after 10 weeks and compared to mice that received SCDIs but did not receive human PBMCs. *CXCL10* knockout was discovered to confer a survival advantage, in addition to known genes like *HLA-A* and *B2M*. CXCL10 is an IFN-induced chemokine, and other members of the family (CXCL9 and CXCL5) were also screen hits, suggesting a common mechanism. *CXCL10* KO SCDIs were generated, transplanted into mice, and graft survival was assessed with or without PBMCs. While a majority of unedited SCDIs were destroyed when mice received PBMCs, *CXCL10* KO SCDI graft survival was significantly improved compared to mice that received unedited SCDIs but did not receive PBMCs. The state-of-the-art Hu-PBL-NSG-MHC^null^ mouse model enables superior PBMC engraftment by preventing human T-cell recognition of murine MHC and the concomitant acute GVHD, but there are also limitations as other aspects of the human immune system may yet prove relevant to understanding the totality of the SCDI-immune interaction(122). Going forward, we anticipate genetic screens to tap deeper into the vast coding as well as noncoding genome for improved survival and immunocompatibility of transplanted cells.

## Brief Conclusion

The application of genome engineering to study and reduce the immunogenicity of SCDI is both an exciting area of inquiry and essential for widespread clinical application. Work is this space is at the vanguard and additional insights will undoubtedly be revealed by future investigations.
